# Effect of Becoming Unemployed on Affordability of Oral Health Care among Australian Adults

**DOI:** 10.1177/23800844241311843

**Published:** 2025-02-21

**Authors:** G. Kaur, G. Tsakos, T. Yap, A. Karahalios, Z. Chen, A. Singh

**Affiliations:** 1 Centre for Epidemiology and Biostatistics, Melbourne School of Population and Global Health, University of Melbourne, Melbourne, Australia; 2 Department of Epidemiology and Public Health, University College London, London, UK; 3 Melbourne Dental School, University of Melbourne, Melbourne, Australia; 4 The University of Sydney School of Dentistry, University of Sydney, NSW 2006

**Keywords:** employment, socioeconomic factors, health care disparities, health equity, health services accessibility, health care costs

## Abstract

**Introduction:**

Oral health care impacts of unemployment are not well understood. This is particularly important as many people, even in high-income countries, lack publicly funded oral health care, creating a financial burden for working-age individuals. This study aims to investigate the short-term effect of becoming unemployed on affordability of oral health care among working-age Australian adults.

**Methods:**

Longitudinal data from the Household, Income and Labour Dynamics in Australia Survey facilitated analysis of those employed in 2016 and examined the effect of becoming unemployed in 2017 on affordability of oral health care in 2018, adjusted for confounding with inverse probability weighting.

**Results:**

Individuals who became unemployed were 2.95 (95% CI, 1.88 to 4.63) times more likely to not receive dental treatment when needed due to a lack of affordability as compared with individuals who remained employed ( *N* = 6,529). On an absolute scale, the aforementioned difference in probability was 9% (95% CI, 3% to 15%).

**Discussion:**

Becoming unemployed had a considerable and immediate negative impact on the affordability of oral health care among working-age Australian adults. Adequate welfare support services are needed to address the immediate financial hardship and consequences that may result due to unemployment.

**Knowledge Transfer Statement:**

Using large population-based cohort data, we established that becoming unemployed hampers the ability to afford oral health care. Our study showed that this impact occurs within a year of unemployment, highlighting how quickly unemployment can create ripple effects for oral health care utilization, cascading into a potential lack of timely treatment or preventive therapies. Our findings highlight the need for adequate welfare support policies to address the immediate financial hardship and consequences that may result due to unemployment.

## Introduction

Access to health care is a fundamental aspect of individual well-being and an essential component of the human right to health (United Nations Committee on Economic Social and Cultural Rights [UNCESCR] 2000). One critical dimension of health care access is the affordability of necessary treatments and services (UNCESCR 2000). Affordability of health care, particularly oral health care, can be affected by socioeconomic status and changes in one’s life circumstances, as most countries do not cover oral health as part of universal health coverage plans and dental treatment is often very expensive (World Health Organization 2022). As a result, many people experiencing social disadvantage do not have access to quality, affordable oral health care, which may contribute to a range of oral health problems, including dental caries and gum diseases (World Health Organization 2022). These conditions are preventable and can have far-reaching consequences for individuals and societies, including pain (Jin et al. 2016), poor nutrition (Jin et al. 2016; Gondivkar et al. 2019), low quality of life (Jin et al. 2016), reduced productivity at school and at work (Listl et al. 2015; Jin et al. 2016), and increased pressure on health care systems (Listl et al. 2015).

Currently oral diseases present a global public health challenge affecting around 3.5 billion people worldwide (Peres et al. 2019) and have accounted for 4.5% of the nonfatal disease burden that affected Australians in 2022 (Australian Institute of Health and Welfare 2023). Public oral health services in Australia are critically underresourced, and this has consequently led to significant waiting lists and overreliance on privately delivered services (Duckett et al. 2019). The exclusion of oral health care from the universal health care scheme in Australia (Medicare) further limits the use of public oral health services, as only a small number of population subgroups (Appendix Table 1; Department of Families, Fairness and Housing 2024) are eligible for modest copayments within the public system. This combination of factors means that most working-age adults in Australia fully fund their oral health expenditure privately. During 2020 to 2021, approximately $11.1 billion was spent on dental services in Australia, of which patients directly paid 59% of the cost, while an additional 20% was funded via private health insurance, which is an additional cost for individuals (Australian Institute of Health and Welfare 2023). Therefore, most people pay for expensive treatments and repeated visits to oral health professionals leading to high out-of-pocket costs.

Employment status is a key indicator of socioeconomic status among working-age adults. However, research on the social determinants of oral health often focuses on income and education, and the role of unemployment, a recognized social determinant of health, remains underexplored. The impact of unemployment on access to oral health care and its affordability extends beyond the immediate loss of disposable income. Unemployment can create other barriers, such as financial strain from dwindling savings (Ryu and Fan 2023), as well as psychosocial stress and poor mental health (Pharr et al. 2012; Modini et al. 2016; Konstantakopoulos et al. 2019). These factors can lead to competing priorities, forcing individuals to forgo oral health care visits in favor of more essential needs, such as housing and food (Quinn et al. 2008). Recognizing the immediate health consequences of unemployment can help shape welfare and public health strategies aimed at detecting early signs of potential long-term health problems and devising timely interventions for population groups at risk. It is important to note that most people, even in high-income countries, do not receive publicly funded oral health care, adding a significant financial burden, particularly for working-age people who often lack public health care coverage altogether (World Health Organization 2022; Australian Institute of Health and Welfare 2023).

Some existing studies have explored the association between unemployment or economic crisis and poor oral health (Sarah et al. 2006; Rodriguez-Alvarez et al. 2019; Urbanos-Garrido 2020; Spinler et al. 2021; Eirey et al. 2022). However, most of the existing evidence relies on cross-sectional data, which limits the ability to draw causal inference. Furthermore, existing studies focus on the utilization of oral health care but overlook the issue of affordability (Sarah et al. 2006; Quinn et al. 2008; Guiney et al. 2011; Rodriguez-Alvarez et al. 2019). If individuals do not find dental care affordable, they might delay or forgo it in favor of more pressing needs. Understanding affordability helps to acknowledge the role of these financial trade-offs and highlights how economic constraints affect decisions about health care spending.

Given the lack of equitable oral health care, studying and addressing the impact of unemployment on the affordability of oral health care becomes even more imperative. Therefore, this study aims to investigate the short-term effect of becoming unemployed on the affordability of oral health care, as measured by the inability to seek dental treatment when needed due to a lack of affordability in working-age Australian adults.

## Methods

The research is reported per the STROBE guidelines (Strengthening the Reporting of Observational Studies in Epidemiology; Appendix Table 2). The Household, Income and Labour Dynamics in Australia (HILDA) Survey is a nationally representative cohort study of Australian households and has collected information regarding the demographic, economic, and health status of Australians annually since 2001 (Watson and Wooden 2012). For this study, we used HILDA data from waves 14 to 18 (years 2014 to 2018) and restricted our sample to participants aged 20 to 54 y in 2016 (wave 16). This upper age cutoff was chosen because the average retirement age of all retirees in Australia is 55.4 y and 55% of Australians aged >55 y are retired (Australian Bureau of Statistics 2018–2019).

The exposure was being unemployed in wave 17. Individuals were categorized as unemployed or employed in wave 17. HILDA categorizes employment status as employed, unemployed, and not in the labor force. Individuals who were missing information on employment status and those who were not in the labor force (i.e., were not actively seeking employment) were excluded from the analysis. Since we focused on transition from employed to unemployed as the exposure, individuals who were unemployed in wave 16 were excluded; therefore, analyses were limited only to those employed in wave 16. Individuals with missing data on employment status in wave 17 were also excluded from the analysis.

Affordability of oral health care was measured in wave 18 by asking “whether you (and your family) have dental treatment when needed.” Those who responded *no* were asked, “Is that because you cannot afford it?” Responses to these 2 questions were combined to derive a categorical variable classifying people who had dental treatment and those who did not have dental treatment due to unaffordability. People who did not have treatment due to other reasons were excluded from the analysis.

**Figure 1.**
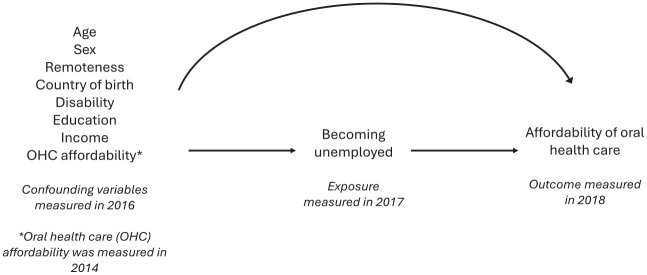
Directed acyclic graph for the association between becoming unemployed and affordability of oral health care.

Age, sex, education, remoteness, country of birth, disability, income, and earlier self-report on affordability of oral health care were considered common exposures to unemployment in wave 17 (exposure) and affordability in wave 18 (outcome) and were included in the models as confounders. All confounding variables, except oral health care affordability, were measured in wave 16. Age was specified as a continuous variable in the models, but age categories (20 to 24, 25 to 34, 35 to 44, and 45 to 54 y) were used for descriptive characteristics. Education was categorized into 3 categories based on the highest education level achieved by the participant: bachelor degree or higher; year 12, certificate, or diploma; and less than year 12. A modified version of the Australian Standard Geographical Classification for remoteness was used and included major city, inner regional, and outer regional/remote/very remote. Participants were categorized as Australian born or overseas born according to their country of birth. Weekly disposable income was categorized into tertiles: lower-, middle-, and higher-income groups. Affordability of oral health care was derived from wave 14, not measured in wave 15 or 16, and categorized similarly to the outcome. Figure 1 shows the directed acyclic graph for the association between being unemployed and affordability of oral health care.

Descriptive analysis was conducted to summarize the outcome and covariates according to the exposure (becoming unemployed vs. staying employed). To adjust for confounding when estimating the effect of exposure on outcome, we used inverse probability weighting (IPW). IPW creates a pseudo population in which confounders are balanced across exposed and unexposed groups (Chesnaye et al. 2022). IPW estimates each person’s probability of being exposed based on confounding variables. To balance on covariate distributions, IPW assigns more weight to individuals who were less likely to be exposed and less weight to those who were more likely to be exposed (Williamson et al. 2012). Next, the probability of the potential outcome (inability to seek dental treatment when needed due to lack of affordability) is estimated once everyone is assigned to stay employed (unexposed group) and, similarly, once everyone is assigned to become unemployed (exposed group). The effect on the absolute difference scale (absolute inequality) is estimated by the average difference between probabilities of the outcome among the exposed and unexposed. The ratio (relative inequality) is estimated by exponentiating the logarithm of the ratio of average probabilities of the outcome among the exposed and unexposed. To account for clustering of individuals by households, households as clusters were identified in IPW estimations, and the standard errors were accordingly adjusted for. Finally, to test the robustness of our findings, we checked and reported the balance of covariates according to exposure categories before and after reweighting. A difference >10% in covariate distribution between unexposed and exposed shows covariate imbalance despite reweighting—hence, the lack of exchangeability (Chesnaye et al. 2022). Additionally, we estimated E-values to examine the amount of residual/unmeasured confounding needed to explain the observed effects (VanderWeele and Ding 2017). All the models were fitted by using the *teffects ipw* command in Stata version 17 (StataCorp).

## Results

**Figure 2.**
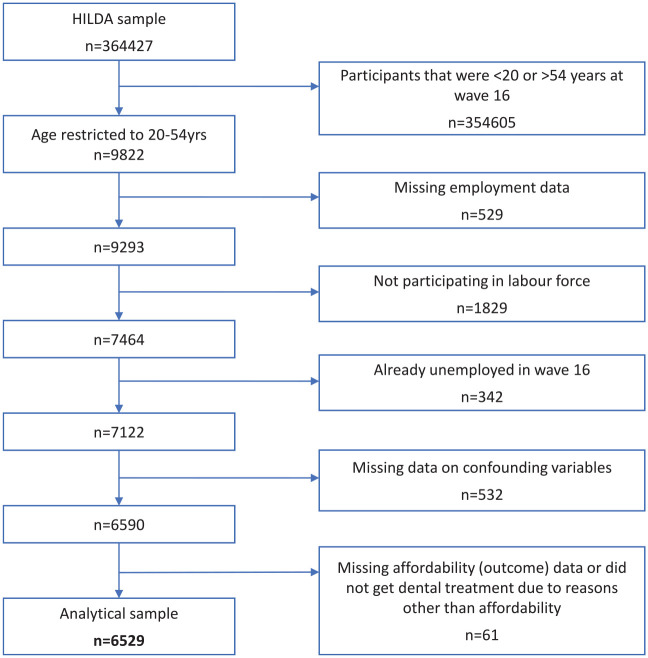
Flowchart of participants in the Household, Income, and Labour Dynamics in Australia (HILDA) Survey in analytic sample.

A sample of 6,529 individuals was used for this analysis. The sample selection process is outlined in Figure 2. Characteristics of the participants in our analysis according to employment status are presented in Table 1. When compared with those who stayed employed, people who became unemployed were more likely to be younger (16.5% vs. 11.9%, 20 to 24 y) male (57.3% vs. 51.7%), have a disability (21.4% vs. 13.2%) possess a lower education level (16.5% vs. 11.8%, less than year 12; 68.0% vs. 52.4%, year 12/certificate/diploma), and fall in the lowest-income tertile (55.3% vs. 32.6%). Differences by remoteness area and country of birth according to employment status were small.

**Table 1.** Demographic Characteristics according to Change in Employment Status (*N* = 6,529).

	Stayed Employed (*n* = 6,426)		Became Unemployed (*n* = 103)	
	No.	%	No.	%
Age, y				
20 to 24	763	11.9	17	16.5
25 to 34	1,947	30.3	24	23.3
35 to 44	1,794	27.9	32	31.1
45 to 54	1,922	29.9	30	29.1
Sex				
Male	3,325	51.7	59	57.3
Female	3,101	48.3	44	42.7
Remoteness area				
Major cities	4,253	66.2	67	65.1
Inner regional	1,489	23.2	22	21.4
Outer regional, remote, very remote	684	10.6	14	13.6
Country of birth				
Australia	5,247	81.7	83	80.6
Overseas	1,179	18.3	20	19.4
Disability				
Yes	849	13.2	22	21.4
No	5,577	86.8	81	78.6
Highest education level				
Bachelor degree or higher	2,297	35.8	16	15.5
Year 12, certificate, diploma	3,369	52.4	70	68.0
Less than year 12	760	11.8	17	16.5
Weekly disposable income, $				
Tertile 1: <1,387.1	2,094	32.6	57	55.3
Tertile 2: 1,387.6 to 2,210.4	2,141	33.3	28	27.1
Tertile 3: 2,211.6 to 18,967.4	2,191	34.1	18	17.5
Baseline affordability of oral health care				
Availed treatment	6,110	95.1	95	92.2
Did not get treatment due to lack of affordability	316	4.9	8	7.8

The raw and weighted standardized differences according to confounders are presented in Appendix Table 3. None of the differences in the distribution of confounding factors and baseline covariates between the exposed and unexposed groups were >10%, suggesting that a balance of covariates was achieved by the IPW. The analysis showed that individuals who became unemployed had 2.95-times higher risk (95% CI, 1.88 to 4.63) of not being able to get oral health care when needed due to a lack of affordability as compared with those who were employed, after accounting for age, sex, education, remoteness, country of birth, disability, income, and affordability of oral health care. On an absolute scale (i.e., risk difference), the probability of not being able to afford oral health care was 9% higher (95% CI, 3% to 15%) in those who were unemployed than those who were employed after accounting for confounding factors (Table 2).

**Table 2.** Total Causal Effect of Becoming Unemployed on Affordability of Oral Health Care (*N* = 6,529).

	Risk Ratio				Risk Difference			
	Unadjusted		Adjusted^a^		Unadjusted		Adjusted^a^	
	Estimate	95% CI	Estimate	95% CI	Estimate	95% CI	Estimate	95% CI
Stayed employed (*n* = 6,426)	Reference	—	Reference	—	Reference	—	Reference	—
Became unemployed (*n* = 103)	4.22	2.80 to 6.35	2.95	1.88 to 4.63	0.15	0.07 to 0.22	0.09	0.03 to 0.15

a Adjusted for age, sex, education, remoteness, country of birth, income, and baseline affordability of oral health care.

The E-value for the overall effect estimate of becoming unemployed on affordability for dental care was 5.35, and the E-values for the low and high confidence intervals were 3.17 and 8.73, respectively. This indicates that at a minimum the effect size of the association of unmeasured confounding with 1) unemployment and 2) the affordability of oral health care needs to be substantially high (at least 3.17 times) to explain away the adjusted risk ratio of 2.95.

## Discussion

We estimated that becoming unemployed increased the risk of not being able to afford oral health care within 1 year by approximately 3-fold (risk ratio, 2.95; 95% CI, 1.88 to 4.63). This study highlights unemployment as a key social determinant that has an impact within a short time frame (1 y) on the affordability of oral health care in working-age adults. It is noteworthy that the risk of being unable to afford oral health care was 9% higher (risk difference, 9%; 95% CI, 3% to 15%) among individuals who became unemployed as compared with those who remained employed, which highlights the substantial impact that becoming unemployed has on the affordability of oral health care.

Our findings expand on cross-sectional findings from other countries (Sarah et al. 2006; Zavras et al. 2016; Urbanos-Garrido 2020; Spinler et al. 2021) and Australia (Pharr et al. 2012) that found that job losses and unemployment are associated with reduced utilization of health care services, which may result in poorer health outcomes. By leveraging the large population-based cohort study data and imposing a temporal ordering between variables, we establish that becoming unemployed has an impact on the inability to afford oral health care. Furthermore, our investigation into the inability to afford oral health care a year after becoming unemployed enabled us to establish the short-term impact of unemployment. Therefore, it signifies how quickly unemployment can create ripple effects for oral health care utilization, potentially cascading into a lack of timely treatment or preventive therapies. The large effect size on absolute and relative scales further stresses how unemployment is a key social determinant for oral health care and population oral health.

Some of the strengths of this study are discussed in turn. First, temporality is an undisputed criterion for causal inference. By using confounding variables from wave 16, exposure from wave 17, and outcome from wave 18, temporal sequence has been maintained among confounders, exposure, and outcome. Second, IPW analysis was used to maximize exchangeability and make robust causal inference (Chesnaye et al. 2022). We also report effect estimates on absolute and relative scales, which provides a comprehensive picture when investigating health inequalities (King et al. 2012). One of the limitations is that the study utilizes self-reported survey data, which may be affected by measurement error. Furthermore, while several potential confounding factors were accounted for in this study, bias may occur due to unmeasured confounding. To partly address this, we have reported E-values quantifying how potential unmeasured confounders may affect the main estimates of interest, and we showed that it would require an excessively strong association between potential confounders and the variables of interest for unmeasured confounding to explain the short-term effect of being unemployed on the lack of affordability for oral health care.

This study contributes findings that are important to consider when drafting policies for improving the oral health of Australians and internationally. Policies need to be geared toward fairer access to curative and preventative oral health services. Since the effect of becoming unemployed on the lack of affordability of oral health care is materializing within a very short time frame, it is important to consider relevant welfare support that is provided to individuals to address immediate financial hardships that may result due to unemployment. Future studies should examine pathways through which unemployment hinders oral health care access. Additionally, further studies are needed to address the long-term effects of unemployment or insecure employment on access to oral health care.

To conclude, becoming unemployed had a considerable and immediate negative impact on the affordability of oral health care among working-age Australian adults. The findings from this study emphasize that employment status is a key social determinant of affordability of oral health care.

## Authors’ Contributions

G. Kaur, contributed to conception, design, data acquisition, analysis, and interpretation, drafted and critically revised the manuscript; G. Tsakos, contributed to conception, design, data analysis and interpretation, critically revised the manuscript; T. Yap, contributed to conception, data interpretation, critically revised the manuscript; A. Karahalios, contributed to data analysis and interpretation, critically revised the manuscript; Z. Chen, contributed to data interpretation, critically revised the manuscript; A. Singh, contributed to conception, design, data acquisition, analysis, and interpretation, critically revised the manuscript. All authors gave their final approval and agreed to be accountable for all aspects of the work.

## Supplemental Material

sj-docx-1-jct-10.1177_23800844241311843 – Supplemental material for Effect of Becoming Unemployed on Affordability of Oral Health Care among Australian Adults Supplemental material, sj-docx-1-jct-10.1177_23800844241311843 for Effect of Becoming Unemployed on Affordability of Oral Health Care among Australian Adults by G. Kaur, G. Tsakos, T. Yap, A. Karahalios, Z. Chen and A. Singh in JDR Clinical & Translational Research
